# The complete chloroplast genome sequence of *Aconitum austroyunnanense* W. T. Wang (Ranunculaceae): a medicinal plant endemic to China

**DOI:** 10.1080/23802359.2019.1700195

**Published:** 2019-12-12

**Authors:** Zi-Dan Cheng, Jun He, Ying-Min Zhang, Cong-Wei Yang, Xiao-Xia Ma, Guo-Dong Li

**Affiliations:** a Faculty of Traditional Chinese Pharmacy, Yunnan University of Chinese Medicine, Kunming, Yunnan, China;; b Kunming Institute of Botany, Chinese Academy of Sciences, Kunming, Yunnan, China

**Keywords:** *Aconitum austroyunnanense*, chloroplast genome, medicinal plant

## Abstract

The complete chloroplast (cp) genome of *Aconitum austroyunnanense* W. T. Wang, a rare and endangered medicinal plant endemic to southwestern China, was sequenced to be 155,818 bp in length, including two inverted repeat (IR, 26,128 bp) regions, one large single-copy region (LSC) and one small single-copy region (SSC) of 86,555 bp and 17,007 bp, respectively. The cp genome has 131 annotated genes, including 85 protein-coding genes, 37 tRNA genes, 8 rRNA genes, and a pseudogene (*ycf1*). The overall GC content of it is 38.1%. Phylogenetic analysis revealed that the cp genome of *A. austroyunnanense* is closely related to that of *Aconitum hemsleyanum.*

*Aconitum austroyunnanense* W. T. Wang, a climbing perennial herb, is a medicinal and poisonous plant endemic to southwestern China, distributed only in the central-southern region of Yunnan Province (Wang 1979). The radix of *A. austroyunnanense* and *Aconitum vilmorinianum* named ‘Caowu’ are of high medicinal value and have been officially listed in Drug Standards of Yunnan Province (1998 edition) for the treatment of traumatic injury, rheumatic joint pain and chills in hands and feet (Li et al. 2017). Due to morphological similarity among *Aconitum* species, and molecular markers for the identification of *Aconitum* species are limited (He et al. 2010), it is very important to carry out phylogeny studies of *Aconitum* plants using chloroplast (cp) genome sequences. To date, the cp genomes of *A. vilmorinianum* and other 18 *Aconitum* species have been reported (Meng et al. 2018). Whereas, more studies on the cp genome are needed for complete molecular identification. In this study, we characterized the complete cp genome sequence of *A. austroyunnanense* to contribute to further molecular identification and phylogenetic position studies of this plant species.

Fresh leaves of *A. austroyunnanense* were collected from Gejiu Country (23°21′N, 103°11′E), Yunnan province, China and voucher specimens (5325010361) were deposited in Herbarium of Yunnan University of Chinese Medicine. Total genomic DNA was extracted using plant DNA (Bioteke Corporation, China). Genome sequencing was performed on an Illumina HiSeq 2500 platform (Illumina Inc., San Diego, CA). A total of 3.1 GB reads were obtained and de novo assembled using NOVOPlasty (Dierckxsens et al. 2017). The complete cp genome was annotated with the online annotation tool GeSeq (Tillich et al. 2017).

The size of the complete cp genome of *A. austroyunnanense* is 155,818 bp (GenBank accession No.: MN635745), containing a large single copy (LSC) region of 86,555 bp and a small single copy (SSC) region of 17,007 bp, which are separated by a pair of inverted repeats (IRs) regions of 26,128 bp. In addition, a total of 131 genes were annotated including 85 protein-coding genes, 37 tRNA genes, 8 rRNA genes, and one pseudogene *yycf1*. The overall GC-content of the whole plastome is 38.1%, while the corresponding values of the LSC, SSC, and IR regions are 36.2, 32.5, and 43.1%, respectively. A total of 62 Simple Sequence Repeats (SSRs) were detected using the online software IMEx (Mudunuri and Nagarajaram 2007). The number of mono-, di-, tri-, tetra-, penta-, and hexa- nucleotides SSRs are 28, 15, 9, 5, 5, and 0, respectively.

To determine the phylogenetic position of *A. austroyunnanense*, a total of 27 species used to construct the phylogenetic tree among the most of Ranunculaceae species, and Berberidaceae species as outgroups. All of the plastomes were aligned using MAFFT v.7 (Katoh and Standley 2013), and the RAxML (Stamatakis 2014) inference was performed by using GTR model with support for branches evaluated by 1000 bootstrap replicates (Figure 1). *Aconitum austroyunnanense* is found to be closely related to species of the *Aconitum* subgenus compared with species of other genera in Ranunculaceae. The complete cp genome of *A. austroyunnanense* will provide a valuable resource for the conservation genetics of this species as well as for the phylogenetic studies of *Aconitum*.

**Figure 1.**
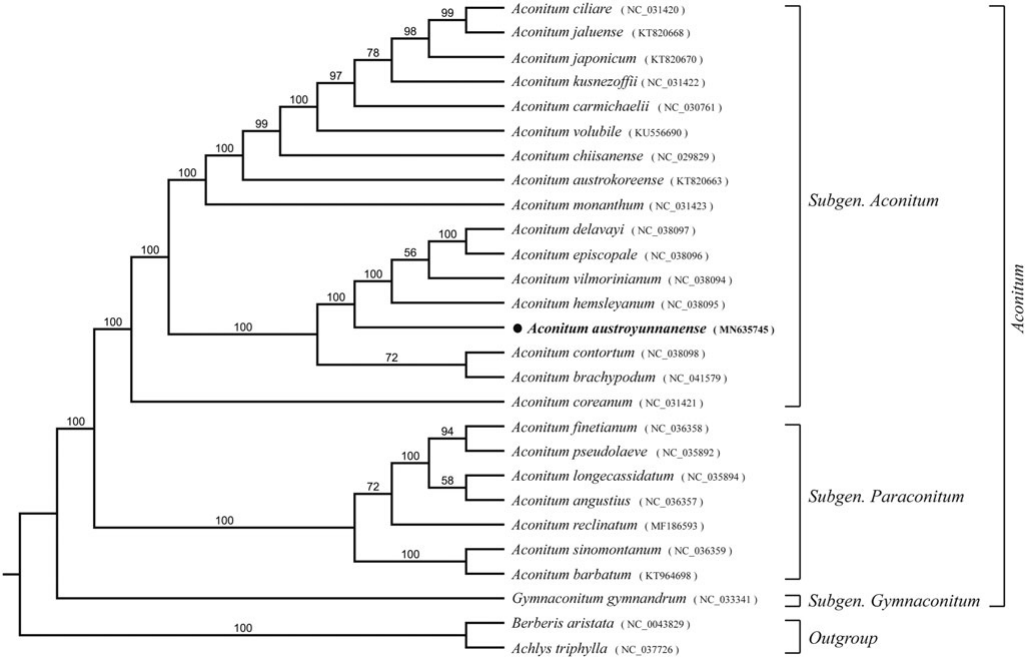
Maximum-likelihood phylogenetic tree inferred from 27 chloroplast genomes. Bootstrap support values >50% are indicated next to the branches.
